# Phytochemical and Structural Portrayal of Barley and Pearl Millet Through FTIR and SEM

**DOI:** 10.1002/fsn3.70120

**Published:** 2025-05-06

**Authors:** Iqra Khalil, Shahid Bashir, Kanza Saeed, Tawfiq Alsulami, Hamad Rafique, Emery Kasongo Lenge Mukonzo

**Affiliations:** ^1^ University Institute of Diet and Nutritional Sciences, Faculty of Allied Health Sciences The University of Lahore Lahore Pakistan; ^2^ University Institute of Food Science and Technology, Faculty of Allied Health Sciences The University of Lahore Lahore Pakistan; ^3^ Faculty of Food Technology and Nutrition Sciences University of Biological and Applied Sciences Lahore Pakistan; ^4^ Department of Food Science & Nutrition College of Food and Agricultural Sciences, King Saud University Riyadh Saudi Arabia; ^5^ College of Food Engineering and Nutritional Science Shaanxi Normal University Xi'an China; ^6^ Land Evaluation and Agro‐Metrology Research Unit, Department of Soil Science, Faculty of Agriculture Research University of Lubumbashi Lubumbashi Democratic Republic of the Congo

**Keywords:** arabinoxylan, Fourier transform infrared spectroscopy, scanning electron microscope, Shahansha pearl millet, Talbina‐21 barley, β‐Glucan

## Abstract

In the present study, *Pennisetum glaucum* variety Shahansha (F1 bajra) and *Hordeum vulgare* variety Talbina‐21 were tested for nutritional profiling, and results exhibited that pearl millet was rich in moisture, fat, carbohydrate, and ash content; whereas, barley had a copious amount of protein and fiber content. Mineral composition analysis showed barley had a plentiful quantity of calcium (43.97 ± 0.06 mg/100 g), phosphorus (350.58 ± 1.39 mg/100 g) and sodium (36.31 ± 0.95 mg/100 g); whereas, pearl millet had higher iron (7.81 ± 0.05 mg/100 g), potassium (306.33 ± 3.2 mg/100 g) and magnesium (135.61 ± 2.19 mg/100 g). Barley also had a high concentration of total phenolic content (204.73 ± 5.5 mg GAE/g) and total flavonoid contents (134.72 ± 4.71 mg QE/g). Antioxidant activity measured through FRAP, ABTS, and DPPH tests indicated distinct antioxidant activity in barley for DPPH (105.72 ± 0.02 mg GAE/g) and ABTS assay (272.08 ± 5.80 μmol TEAC/100 g), while pearl millet showed stronger activity for FRAP assay (5.22 ± 0.04 TE/g). Using Fourier Transform Infrared Spectroscopy (FTIR), functional groups in the flours were identified, and Scanning Electron Microscopy (SEM) revealed that barley had smaller, spherical granules with smooth edges, while pearl millet had a rough, wrinkled surface with hollow cylindrical morphology. The compositional analysis of the flours revealed the presence of various sugars, proteins, ferulic acid, uronic acid, and dietary fiber components (arabinoxylan & β‐glucan). Conclusively, millet and barley possess pronounced phenolic composition, high antioxidant potential, and dietary fibers like arabinoxylan & β‐glucan offer substantial biological efficacy in human health interventions.

## Graphical abstract

In the present study, pearl millet variety Shahansha and barley variety Talbina‐21 were tested for nutritional profiling, and results exhibited that pearl millet was rich in moisture, fat, carbohydrate, and ash content, whereas barley had a copious amount of protein and fiber content. Mineral composition analysis showed that barley had a plentiful quantity of calcium, phosphorus, and sodium, whereas pearl millet had higher iron, potassium, and magnesium. Barley also had a high concentration of total phenolic and flavonoid contents.

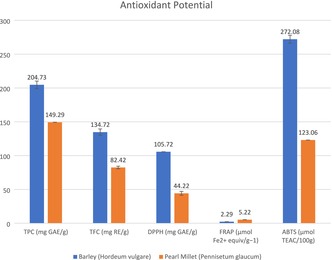

## 1 Introduction

Pearl millet ( *P. glaucum* ), grown mostly for food and fodder in India and Africa, and as a forage crop in the Americas. It is an extensively grown small grain and is rich in essential minerals and dietary fiber (Gull et al. 2017). Millet is considered to be a high‐quality grain due to its diverse phenolic profile and antioxidant potential (Jagdale et al. 2023). This crop is widely grown in 7 major states of India, covering a total area of 7.95 million hectares, 8.90 Mt. Each year, globally over 26 million hectares are planted with it, 11 million in South Asia and West Africa, and 2 million in Brazil and Eastern/Southern Africa (Gull et al. 2014; Pattanashetti et al. 2016). According to ICRISAT (2016), it is grown on over 31 million hectares worldwide, whereas Pakistan grows pearl millet ( *P. glaucum* ) on approximately 0.50 million hectares, yielding 0.33 million tonnes (GOP 2015) (Arshad et al. 2023). As per the latest available statistics, the Directorate of Agriculture, Crop Reporting Services Lahore, Punjab, Pakistan, states that the crop area of pearl millet ( *P. glaucum* ) in Punjab grew by 3.58% in 2014 (423.20 thousand hectares; 273.6 thousand tonnes production). The primary manner in which it is eaten is in the form of thick porridge (toh). However, it is also ground into flour for making couscous, fermented meals like kisra and gallettes, nonalcoholic drinks, and snacks in addition to unfermented bread and cakes like roti (Satyavathi et al. 2022). *P. glaucum* variety Shahansha (F1 bajra) is a hybrid pearl millet variety grown in arid and semi‐arid regions of Asia and South Africa, known for its high yield, drought, and disease resistance. This variety is rich in protein, fiber, and minerals like iron and magnesium (Gate 2017).

Globally, barley ( *H. vulgare* ) production in 2016–17 was 145 million metric tonnes, and the top producers were France, Germany, Canada, Australia, Russia, and Ukraine. The strong and versatile grain known as barley is currently produced in more than 100 nations throughout the world (Suman and Sreeja 2019). It is additionally cultivated in several tropical places while being mostly farmed in temperate nations; as feed for cattle, malt products, and human consumption. Barley plays a vital role in the global food chain (Shaveta et al. 2019). Barley ( *H. vulgare* ) has a starch percentage ranging from 53% to 67%, dietary fiber content ranging from 14% to 25%, and crude protein level ranging from 9% to 14%. It also contains 3%–4% crude fat, 2%–3% ash and 1%–7% low molecular weight carbohydrates (LMWC). The Talbinah‐21 variety of barley is the first hull‐less variety prepared in the year 2021 at the Wheat Research Institute (WRI), Ayub Agricultural Research Institute (AARI), Faisalabad, Punjab‐Pakistan. In 2021, this variety was released in irrigated and moisture stress regions of Punjab‐Pakistan. The Punjab Seed Council, Pakistan, approved this variety in 2021 for general cultivation in Barani areas. This high‐yielding variety enhances the genotypic diversity and will cater to the local demands of hull‐less barley (Ahmad et al. 2022).

Among cereal crops, barley ( *H. vulgare* ) grains have the best antioxidant properties (high‐glucans, resistant starch, and low glycemic index) (Punia Bangar et al. 2022). Research on the possible use of dietary fiber (DF) due to its beneficial functional and therapeutic effects is accumulating as public knowledge of diet and health care grows. Overall, dietary fiber is made up of cellulose, dextrin, pectin, lignin, β‐glucan, and xylooligosaccharides in addition to non‐starch polysaccharides. Because of these components, dietary fibers have high viscosity in aqueous solution and they have a significant impact on the cereal grain's functional utility (milling, baking, feeding animals) (Luithui et al. 2019).

Arabinoxylans have phenolic moieties like ferulic acid, p‐coumaric acid, and etc. in their molecular structures, which give them antioxidant properties. In fact, ferulic acid (FA), one of the phenolic acids of AX, has strong antioxidant potential. During redox processes, arabinoxylans can donate both electrons and hydrogen atoms. Their antioxidant potential is greatly affected by their dietary sources, replacement pattern, amount of xylose substituted, and ferulic acid content (Chen et al. 2019). The primary role of antioxidants is to postpone or stop the oxidation that free radicals cause (Wang et al. 2020). Due to its antioxidant capacity, arabinoxylan decreased the incidence of diabetes and colorectal cancer (Chen et al. 2019). For arabinoxylan to exert these health benefits, two processes may be necessary. They consist of: (a) water‐extractable arabinoxylans that hinder intestinal α‐glucosidase and glucose transporter noncompetitively, lowering postprandial blood glucose levels; and (b) arabinoxylans that neutralize dietary free radicals that trigger the onset and progression of chronic illnesses by donating electrons or hydrogen atoms as they pass through the digestive tract (Bader Ul Ain et al. 2018). Bijalwan et al. (2016) found that feruloyl arabinoxylans had exceptionally potent antioxidant activity in their study on water‐soluble, non‐starch polysaccharides from malted and native finger millet. Ferulic acid present in millet explains the reason of several folds (4.9–1400) higher activity than the predicted potential. Sulfated polysaccharides, on the other hand, could be the reason of thousand‐fold higher activity (901–5000).

Fourier Transform Infrared Spectroscopy (FTIR) and Scanning Electron microscopy (SEM) are advanced analytical procedures used to investigate structural and chemical characteristics of materials (Przybył et al. 2021). FTIR can efficiently detect and characterize polysaccharides (arabinoxylans, Starch, cellulose), proteins (gluten), and phenolic acids (ferulic acid) (Sztupecki et al. 2023). In polysaccharides, C‐O and C‐C stretching vibrations are associated peaks that are detected (Liu et al. 2021). In proteins, C=O and N‐H stretching vibrations demonstrate amide I and amide II bands (Singh et al. 2021). In lipids, C‐H and C=O stretching vibrations can be identified (Kalaimani et al. 2021). In phenolic acids, C=C and O‐H stretching vibrations are detected (Bensemmane et al. 2021).

To study microstructures and surface morphology of foods, high‐resolution SEM is used (Pipliya et al. 2024). In cereal grains, SEM provides detailed images of surface and internal structures. This technique has the potential to reveal surface characteristics of outer bran, aleurone, and endosperm layers (Langton and Gutiérrex 2021). SEM can visualize physical characters like pores, cracks, shape, size, and morphology of starch granules (AnushaDas and Mazumder 2024). Furthermore, this technique helps to provide a deep insight regarding structural arrangement of arabinoxylans, polysaccharides, and cellulose in the cell wall (Wu et al. 2024).

The objective of this study was to determine the nutritional profile (moisture, protein, ash, fiber, fat, nitrogen free extracts, calcium, iron, phosphorus, sodium, potassium, magnesium) and phytochemical profile (TPC, TFC), antioxidant potential (DPPH, ABTS, FRAP) and structural analysis of powdered pearl millet ( *P. glaucum* ) and barley ( *H. vulgare* ) using Fourier Transform Infrared Spectroscopy FTIR and Scanning Electron Microscopy SEM.

## 2 Materials and Methods

The study was carried out at the Institute of Diet and Nutritional Sciences, The University of Lahore. In this study, Shahansha pearl millet ( *P. glaucum* ) and Talbina‐21 barley ( *H. vulgare* ) powder were analyzed to determine their biochemical and nutritional profiles.

### 2.1 Procurement of Raw Material

Barley (*H. vulgare L*.) and pearl millet seeds ( *P. glaucum* ) were procured from Green Gold Agri Seeds (Pvt), Faisalabad. Barley ( *H. vulgare* ) and pearl millet seeds (*P. glaucum*) were dried by a hot air dryer at 70°C for 24 h. Then, both samples were pulverized with a blender for 30 s to produce flour. Flour was screened through a 35‐mesh sieve and stored at −20°C until used. The reagents and chemicals of analytical grade were obtained from the laboratories of the Department of Nutritional Sciences, The University of Lahore. All raw ingredients were stored at room temperature.

### 2.2 Chemical Composition

The chemical analysis of both kinds of cereal barley ( *H. vulgare* ) and pearl millet ( *P. glaucum* ) was performed to determine the level of NFE as well as moisture, ash, crude fiber, crude fat, and crude protein, according to their corresponding methods as defined in AACC (2009).

### 2.3 Moisture Content

Moisture contents of samples were determined by hot air oven using method number 44–15.02 given in AACC (2009). At first, samples were weighed by digital weight balance and taken into the China dishes. The china dishes were placed in the hot air oven at 105°C for 24 h. After the time period of 24 h, the dried samples were removed from the hot air oven and put into the desiccator to avoid moisture absorption from the environment. Three times, samples were dried until constant readings were obtained. The given formula was used to calculate the moisture content of the samples:

$$\text{Moisture}\%=\frac{\text{before drying Sample}\;\mathrm{wt}.–\text{after drying sample}\;\mathrm{wt}.}{\;\text{before drying sample}\;\mathrm{wt}.}\times 100$$

### 2.4 Ash

Ash content of samples was determined by muffle furnace using method number 08–03.01 given in AACC (2009). For the determination of ash content, weighed samples were put into the dried weighed crucibles, and samples were gently charred over a low flame until smokeless residues were attained. Crucibles were placed in the muffle furnace at 525 to 550°C for 5 h until the grayish‐white ash remnants were obtained in the crucible. The weight of the sample after ashing was measured. The given formula calculated the ash percentage.

$$\mathrm{Ash}\%=\frac{\text{weight of sample after ashing}}{\text{weigh of sample before ashing}}\times 100$$

### 2.5 Crude Protein

The crude protein of samples was determined by Kjeldahl apparatus following the procedure described in method number 46–10.01 of AACC (2009). The weighed samples, along with one digestion tablet and 25 mL of concentrated H_2_SO_4_, were digested in the digester until the light green color was visible. After that, the digested sample was diluted to make the 250 mL volume. 10 mL of digested sample, along with the 10 mL of 40% NaOH, were taken in the distillation assembly. 4% boric acid, along with phenolphthalein indicator (2–3 drops) was added in a separate beaker and placed in the distillation assembly. The light golden color of samples was obtained at the end of distillation; after that, the samples were titrated by using 0.1 N H_2_SO_4_ until a light pink color was attained. A constant factor of 6.25 was multiplied to find the percentage of crude protein.

$$\text{Nitrogen}\%\frac{\text{Volume of}\;o.1N\;H2\mathrm{SO}4\times \text{Volume of dilution}\;\left(0.0014\right)\;}{\text{Weight of sample}\times \text{Volume of dilutted sample}}\times 100$$

$$\text{Crude protein}\%=\text{Nitrogen}\;\left(\%\right)\times 6.25$$

### 2.6 Crude Fat

Using the Soxhlet apparatus and the procedures outlined in AACC (2009) method number 30–10.01, the crude fat content of both samples was determined. Initially, a 5 g sample of each variety that had been oven dried was wrapped in filter paper. Pre‐weighed samples were placed inside a thimble, and n‐hexane was used to extract the fat from the sample multiple times until all of the fat had been completely removed. An evaporator was utilized in order to recover the n‐Hexane. The crude fat % was determined using the formula given:

$$\text{Crude}\;\mathrm{fat}\%=\frac{\text{Weight of sample before extraction of}\;\mathrm{fat}–\text{Weight of sample after extraction of}\;\mathrm{fat}\;}{\text{Weight of sample after extraction of}\;\mathrm{fat}\;}\times 100$$

### 2.7 Crude Fiber

Samples for crude fiber analysis were gathered by following the procedures outlined in AACC (2009) Method No. 32–10. The amount of crude fiber in 2 g of a sample devoid of fat and moisture was computed. Following a 30‐min boil with 1.25% H_2_SO_4_, the samples were filtered and cleaned. After being heated in 1.25% NaOH for 30 min, these samples were filtered and cleaned. After the residue was produced, it was weighed after being dried at 130°C for 2 h. The dried residue was reweighed, followed by ashing, cooling, and reweighing. Using the following formula, the crude fiber was calculated:

$$\%\text{Fiber}=\frac{\text{loss in weight}\;\mathrm{on}\;\text{ignition}-\text{blank}}{\text{Weight of samples}}\times 100$$

### 2.8 Nitrogen Free Extract (Total Carbohydrates)

By subtracting the percentages of crude fat, crude protein, crude fiber, and total ash from 100 as provided by the AACC (2009), the nitrogen‐free extract (NFE) was calculated.

### 2.9 Mineral Content

Mineral contents, including Na, K, Ca, and Mg, were ascertained from samples of pearl millet ( *P. glaucum* ) and barley ( *H. vulgare* ) using the AOAC (2016) technique. One gram of the material was digested at temperature of 180°C–200°C using 10 mL of a 7:3 nitric acid: perchloric acid mixture until transparent content was formed. Double‐distilled water was used to dilute the combination until a final volume of 100 mL was achieved. An Atomic Absorption Spectrophotometer (Model: Varian, AA‐240, Victoria, Australia) driven by an air‐acetylene flame was used to measure the concentration of mineral components in the diluted sample.

### 2.10 Phytochemical Screening

#### 2.10.1 Total Phenolics

Using the Folin–Ciocalteu assay, the sample's total phenolic content was evaluated by following the procedure followed by Rasheed et al. (2023). Briefly, 1 g of sample of each variant was mixed with 10 mL of 1% acidified methanol (hydrochloric acid: water solution in 4:1(v/w)), vortexed for 1 min, and homogenized with an orbital stirrer for 18 h at 4°C. Then, the mixture was centrifuged at 10,000 rpm for 15 min, and the supernatant was extracted and mixed with Folin–Ciocalteu reagent and placed in the dark. The absorbance was measured at 760 nm, and the results were expressed as mg of gallic acid equivalent (mg GAE/g DM) and quantified using the protocol defined by Slama et al. (2020).

#### 2.10.2 Total Flavonoids

Total Flavonoid content in the barley ( *H. vulgare* ) and pearl millet ( *P. glaucum* ) samples was determined by the AlCl_3_ coulometric method used by Ge et al. (2021) with little modifications. A 10 mL volumetric flask containing 4 mL of distilled water was supplemented with an aliquot of 1 mL of sample extract in methanol. Initially, 0.3 cc of 5% sodium nitrite was introduced to the flask. 3 cc of 10% AlCl_3_ was added to the flask after 5 min. 2 mL of 1 M sodium hydroxide was added to the liquid at the 6‐min mark. 2.4 mL of distilled water was added right away, bringing the total amount of the combination up to 10 mL, and everything was thoroughly mixed. The pink‐colored mixture was attained, and its absorbance was measured at 510 nm in comparison to a blank using a Microprocessor UV–Vis spectrophotometer‐2371. A calibration curve created for standards of quercetin (10 to 100 g/mL) was used to quantify the flavonoid levels, and the result was represented as mg of quercetin equivalent per gram of extract.

### 2.11 Determination of Antioxidant Activity

#### 2.11.1 Ferric Reducing Antioxidant Power (FRAP) Assay

The experiment was performed according to the method given by Omoba et al. (2015). Briefly, Extracts (50 μL) were mixed with 700 μL of ferric‐TPTZ (2,4,6‐tripyridyl‐s‐triazine) reagent prepared by mixing 300 mmol/L acetate buffer (pH 3.6), 10 mmol L^−1^TPTZ in 40 mmol L^−1^ HCl, and 20 mmol/L FeCl_3_ in a 10:1:1 ratio and measured at 593 nm. FeSO_4_ 7H_2_O was used as a standard, and a calibration curve was prepared with six concentrations between 1 and 1000 μmol g^−1^ and antioxidant power assessed through FRAP was expressed as μmol Fe^2+^equiv/g^−1^.

#### 2.11.2 2,2‐Diphenyl‐1‐Picrylhydrazyl (DPPH) Free Radical Scavenging Activity

For determination of 2,2‐diphenyl‐1‐picrylhydrazyl (DPPH) free radical scavenging activity of pearl millet and barley, the method used by Siroha et al. (2016) and Zhou et al. (2021) was followed, with slight modifications. Briefly, 50 μL of the extract was mixed with 1 mL DPPH solution, and the mixture was shaken well and incubated for 30 min in a dark place. The inhibition percentage was determined by the absorbance at 517 nm of the DPPH solution mixed with 80% methanol. For the DPPH assay, the standard antioxidant Trolox was used.

#### 2.11.3 2,2′‐Azino‐Bis‐3‐Ethylbenzothiazoline‐6‐Sulfonic Acid (ABTS) Assay

5 mmol of pure ABTS was dissolved in 5 mM of phosphate‐buffered saline at a pH of 7.4 to create a final solution. The fluid was filtered by passing through the activated MnO using Whatman No. 1 filter paper. To facilitate effective radical generation, the filtered solution was given a dark period for 12 h. Then, 0.2 mm syringe filters were used to filter the incubated solution, which was then stored in a light‐protected area. The average of two readings of the blank sample was taken to generate a standard calibration curve. For each run, 50, 100, 150, 200, and 250 mmol/L of the standard antioxidant Trolox was used. In the 96‐well microplate with 190 μL of ABTS and 10 μL of the prediluted sample, the absorbance was measured at 620 nm following the inhibition period of 20 min (Salman et al. 2013).

### 2.12 Extraction of Arabinoxylan and β‐Glucan

#### 2.12.1 Enzymatic Extraction

With minor adjustments, the technique of Kim et al. (2017) was utilized to extract arabinoxylan and β‐glucan from pearl millet ( *P. glaucum* ) and barley ( *H. vulgare* ). The extraction was carried out at room temperature. Powdered barley ( *H. vulgare* ) and pearl millet ( *P. glaucum* ) were cooked in 85% ethanol for 2 h in order to remove volatile oils, oligosaccharides, and other minor organic compounds. Following that, samples were centrifuged for 15 min at 4000 RPM and treated with water (1:10 w/v) for 15 min at 25°C. To counteract the impact of β‐glucan, samples underwent three rounds of hot water treatment. The residues were then separated from the supernatant using centrifugation. Following a 15‐min centrifugation at 4000 rpm, 2 mol/L HCl was added to the obtained supernatant to bring its pH down to 6.5. Next, starches were eliminated from the supernatant by hydrolyzing it with α‐amylase for 3 h at 95°C.

### 2.13 Structural Analysis

#### 2.13.1 Fourier Transform Infrared Spectroscopy (FTIR)

The Fourier Transform infrared spectroscopy (FTIR) method was used to investigate the functional components of AX in samples of pearl millet and barley (Shimadzu‐8400). For the purpose of detecting the spectrum for functional components, which were found to be between 400 and 4000 cm^−1^, the scanning sample was essentially positioned and subjected to infrared radiation, and while a computer‐connected detector continuously scanned the sample to generate spectral data. The primary goal of AX characterization is to investigate any functional chemicals that may be present in the sample. The procedure employed by Rasheed et al. (2023) is followed in order to characterize the material using FTIR.

#### 2.13.2 Scanning Electron Microscope (SEM)

Applying the technique outlined by Raza et al. (2022), a scanning electron microscope (SEM) was used to examine the microscopic appearance of AX isolated from barley and pearl millet. Scanning electron microscopy (SEM) (FEI Nova nanosem 450, Regen Microscopy, Lemesos, Cyprus) was a useful method for characterizing the morphology of AX and β‐glucans. The cube series manufacturer, Craft, provided the scanning electron microscope. The sample was positioned on stubs at a 5‐kV accelerating voltage, and the micrographs of the sample were analyzed to comprehend its structural characteristics.

#### 2.13.3 Statistical Analysis

All resulting data was analyzed statistically using software StatPlus to test the significance level via the Completely Randomized Design (two‐way ANOVA) described by Montgomery (2017).

## 3 Results and Discussion

### 3.1 Hysicochemical Analysis

#### 3.1.1 Chemical Composition of Barley and Pearl Millet

The chemical composition of dried barley ( *H. vulgare* ) and pearl millet ( *P. glaucum* ) was determined. All analyses were done in triplicate and the results were reported on a dry matter (DM) basis. Mean results for each component moisture%, ash%, protein%, fat%, fiber%, and NFE% are described below in Table 1. Different letters (a, b) indicate statistically significant differences between the means of the Pennisetum glaucum variety Shahansha (F1 bajra) and Hordeum vulgare variety Talbina, whereas, same letter means no significant difference. The statistical data demonstrated that both cereals showed highly substantial differences. Talbina‐21 (Barley) has significantly higher Moisture, Protein, Fiber. Shahansha (Pearl Millet) has significantly higher Ash, Fat, NFE (Carbohydrates). Moisture content plays a vital role in impacting taste, shelf life, safety, texture, and overall quality of food. Moisture in a product determines the density, viscosity, physical appearance, and composition of the product, as it simply refers to the amount of water present in any product (Alemu 2023). Moisture content plays a crucial role in controlling microbial load, thereby preventing food spoilage (Chitrakar et al. 2019). Moisture content is a basic parameter for the classification of all food products (Joardder et al. 2019).

**TABLE 1:** Chemical composition of Barley and Pearl millet.

Chemical composition %	Barley ( *H. vulgare* ) Talbina‐21	Pearl millet ( *P. glaucum* ) Shahansha
Moisture	12.80 ± 0.13^a^	11.20 ± 0.5^b^
Ash	2.29 ± 0.01^b^	2.68 ± 0.02^a^
Protein	13.60 ± 0.02^a^	11.15 ± 0.01^b^
Fat	2.75 ± 0.03^b^	4.87 ± 0.21^a^
Fiber	4.71 ± 0.01^a^	2.92 ± 0.03^b^
NFE	63.86 ± 0.10^b^	67.20 ± 0.14^a^

*Note:* The values are expressed as mean ± standard deviation of triplicate values.

The mean values for moisture content of barley ( *H. vulgare* ) were 12.80% ± 0.13%, while in pearl millet ( *P. glaucum* ), the moisture content was 11.20% ± 0.50%. However, the mean values for ash content of barley and pearl millet were 2.29% ± 0.01% and 2.68% ± 0.02%, respectively. Ash refers to the inorganic residue remaining after either ignition or complete oxidation of organic matter in a foodstuff. The ash content generally represents the concentration of mineral contents present in the given product (Hait et al. 2023). Soil composition and characteristics, genetic makeup, climatic conditions, and milling process are predominant factors influencing the ash content of cereal flours (Kanwal et al. 2023). Protein is an important constituent of our diet and plays a vital role in the human body, such as tissue repair and maintenance, muscle building, cognitive development, and etc. (Shevkani and Chourasia 2021).

Protein constituents of any food product are critically important because of their significant impact on nutritional value and functionality. In our current study, the mean values for protein, fat, fiber, and NFE content of barley ( *H. vulgare* ) and pearl millet ( *P. glaucum* ) were (13.60% ± 0.02%, 11.15% ± 0.01%), (2.75% ± 0.03%, 4.87% ± 0.21%), (4.71% ± 0.01%, 2.92% ± 0.03%), (63.86% ± 0.10%, 67.20% ± 0.14%) respectively. Fat is a key component that determines the baking quality of flour (Yazar and Rosell 2023) and serves as a source of energy and shelf life regulator (McClements and Decker 2018). Pearl millet ( *P. glaucum* ) demonstrated higher fat content as compared to barley ( *H. vulgare* ). The crude fiber content was found to be slightly higher in barley ( *H. vulgare* ) compared to pearl millet ( *P. glaucum* ). Genetics, climate, and soil fertility conditions may all contribute to the variances in chemical makeup.

Previously, a study conducted by Alijošius et al. (2016) in Lithuania comparing six spring and six winter barley ( *H. vulgare* ) varieties found that the spring varieties had greater crude protein levels (10.35%–12.38% DM), with variety Michelle having the highest amount. In both types, the ranges for crude fat and crude ash were 1.09%–2.00% DM, 1.94%–2.40% DM, and 65.45%–69.08% DM for NFE, and their findings are consistent with the outcomes of our current study about the chemical makeup and antinutritional aspects of barley ( *H. vulgare* ). A different study conducted by Kulthe et al. (2016) assessed the nutritional makeup of pearl millet. Upon analysis, the grain was found to contain moisture, ash, fat, protein, crude fiber, and carbs (11.21%–12.43%, 2.05%–2.72%, 5.14%–5.96%, 10.97%–11.65%, 2.07%–2.63%, 66.49%–68.85%) respectively. These findings were similar to those of our current investigations.

Presently, Hussain et al. (2021) investigated the nutritional and functional makeup of four barley ( *H. vulgare* ) varieties from various Gilgit‐Baltistan locations. The nutritional profiles for crude starch, fiber, protein, ash, and fat were, in that order, 56.3%–50.80%, 16.50%–11.73%, 16.20%–11.53%, 2.8%–2.1%, and 2.63%–1.63%, respectively. The proximate composition values of barley grown in Pakistan are determined to be within the results of the investigation's range.

#### 3.1.2 Determination of Mineral Content

Minerals are elements that an organism needs as an essential ingredient to carry out vital functions that are necessary to sustain healthy life. The mineral composition of barley and pearl millet is shown in Table 2. mean values for calcium, iron, phosphorus, sodium, potassium, and magnesium in the barley powder were (43.97 ± 0.06 mg/100 g), (6.49 ± 0.03 mg/100 g), (350.58 ± 1.39 mg/100 g), (36.31 ± 0.95 mg/100 g), (142.53 ± 3.18 mg/100 g), and (59.27 ± 1.65 mg/100 g), respectively. The subscript letters (a,b) indicates statistically significant difference (*p* < 0.05) between Barley and and Pearl Millet for each mineral. Peral Millet (Shahansha) is richer in iron, potassium and magnesium and Barley (Talbina‐21) is richer in phosphorus, sodium and calcium. The findings indicated that barley flour had a higher concentration of phosphorus. Phosphorus is necessary for bone health, energy metabolism, cellular structure, DNA synthesis, and acid–base balance, among other processes that are vital to general body function and wellbeing. Similarly, potassium, another abundant mineral found in barley, is necessary for the body's electrical and cellular processes as well as for maintaining the ionic balance of the body (Udensi and Tchounwou 2017).

**TABLE 2:** Mineral content in Barley and Pearl millet.

Mineral (mg/100 g)	Barley ( *H. vulgare* ) Talbina‐21	Pearl millet ( *P. glaucum* ) Shahansha
Calcium	43.97 ± 0.06^a^	43.69 ± 0.6^b^
Iron	6.49 ± 0.03^b^	7.81 ± 0.05^a^
Phosphorus	350.58 ± 1.39^a^	313.62 ± 10.2^b^
Sodium	36.31 ± 0.95^a^	11.06 ± 0.10^b^
Potassium	142.53 ± 3.18^b^	306.33 ± 3.2^a^
Magnesium	59.27 ± 1.65^b^	135.61 ± 2.19^a^

*Note:* The values are expressed as mean ± standard deviation of triplicate values.

However, mean values for the mineral content of pearl millet ( *P. glaucum* ) showed that the amount of calcium (43.69 ± 0.6 mg/100 g), iron (7.81 ± 0.05 mg/100 g), phosphorus (313.62 ± 10.2 mg/100 g), sodium (11.06 ± 0.10 mg/100 g), and potassium (306.33 ± 3.2 mg/100 g), respectively. However, barley and pearl millet flour are also great suppliers of calcium, which is required for controlling glandular secretions, mediating vascular contraction, and regulating muscle contraction (Melaku and Elias 2023). In addition to helping to move ions across the cellular membrane and activate specific enzymes, calcium is essential for cells to sustain a regular heartbeat (Bootman and Bultynck 2020). In contrast, barley flour ( *H. vulgare* ) and pearl millet ( *P. glaucum* ) contain the lowest amounts of iron, sodium, and magnesium, all of which are essential for maintaining electrolyte balance, supporting neuronal function, and facilitating oxygen transport; all of the functions necessary to promote general health and strength.

In a previous study, Dobhal and Awasthi (2021) explored the nutritional and mineral content of barley flour, and the results of our current study were closely comparable to their research. Gull et al. (2016) found that the mineral content and functional properties of millet flour ( *P. glaucum* ) were as follows: for calcium, zinc, iron, sodium, and potassium, the results were (109.2–139.2 mg/100 g), (0.73–4.2 mg/100 g), (1.18–8.7.0 mg/100 g), and (15.03–17.36 mg/100 g), respectively.

#### 3.1.3 Antioxidant Activity of Barley and Pearl Millet

Antioxidant activity of phenolic compounds derived from vegetables, cereal, fruits, etc. is frequently assessed by measuring the DPPH radical's ability to be scavenged. The basic goal of antioxidants is to prevent oxidative lipid breakdown and preserve food quality. Since DPPH is a stable radical, it is frequently employed to assess its capacity to scavenge free radicals (Gulcin and Alwasel 2023). The free radical scavenging potential of phenolic compounds is based on the number of antioxidants lowering power towards DPPH and FRAP. FRAP shows that the extract can convert Fe3^+^ to iron Fe^2+^ while DPPH provides electrons to prevent lipid peroxidation (Adeniran 2018).

Mean values of total phenolic content and flavonoid contents in barley ( *H. vulgare* ) and pearl millet flour ( *P. glaucum* ) were (204.73 ± 5.5 mg GAE/g, 149.29 ± 0.02 mg GAE/g, respectively), and (134.72 ± 4.71 mg QE/g, 82.42 ± 1.92 mg QE/g, respectively). Meanwhile, mean values of ABTS content in barley flour ( *H. vulgare* ) were (272.08 ± 5.80 μmol TEAC/100 g) and in pearl millet flour ( *P. glaucum* ) were (123.06 ± 0.07 μmol TEAC/100 g), respectively. Previously, Siroha et al. (2016) investigated the antioxidant potential of Indian pearl millet cultivars and observed notable variations in flavonoid content, antioxidant activity, and total phenolic content. Varietal GHB‐732 demonstrated the maximum amount of total phenolic content (3137 μg GAE/g) and DPPH activity (46.7%), but variety HC‐10 displayed the highest number of total flavonoids (2484 μg ce/g). Similarly, Mareček et al. (2017) investigated the antioxidant capacity of various barley cultivars, and their findings were comparable to our present findings.

**FIGURE 1:**
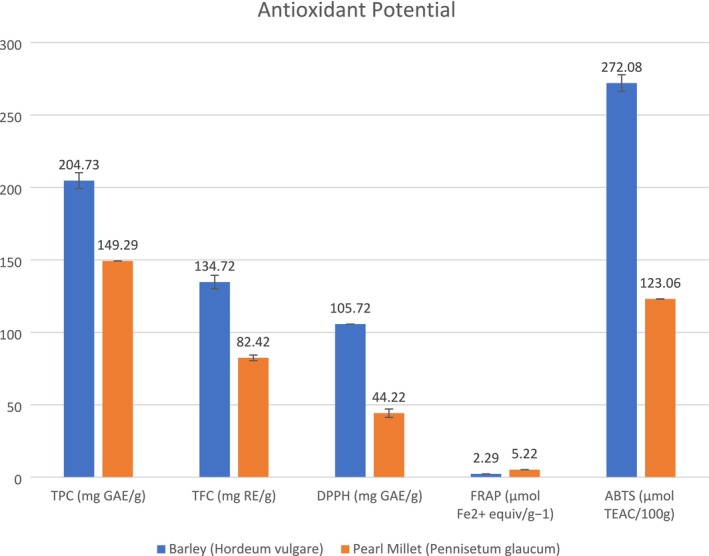
Antioxidant Potential of Barley and Millet.

Mean results of the antioxidant activity of barley and pearl millet with methanolic extract by DPPH assay were (105.72 ± 0.02 mg GAE/g), (44.22 ± 2.88 mg GAE/g), respectively, and for FRAP assay were (2.29 ± 0.03 TE/g), (5.22 ± 0.04 TE/g), respectively, as shown in Figure 1. However, flavonoids are one of the most significant components of natural extracts to consider when assessing the therapeutic value of plants and cereals, and phenolic compounds are a class of chemical molecules that are widely found in nature and have a variety of antioxidant activities (Nwozo et al. 2023). Using the Folin–Ciocalteu reagent method, the total phenolic content of three replicate extracts was determined and expressed as GAE/g dried extract of barley ( *H. vulgare* ) and pearl millet flour.

#### 3.1.4 Structural Analysis

##### 3.1.4.1 FTIR of Barley Samples

**FIGURE 2:**
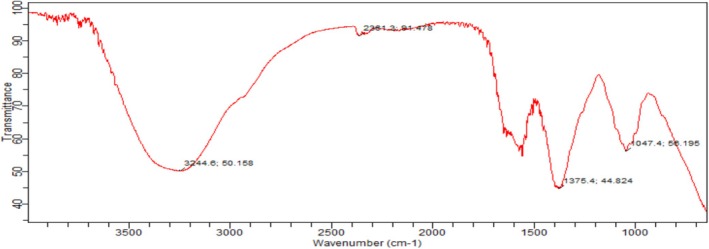
Graphical Representation of FTIR Spectra of Barley flour ( *H. vulgare* ).

Vertex 70 ATR‐FTIR spectrometer was used to analyze the barley ( *H. vulgare* ) FTIR spectrum (Bruker, UK) with a resolution of 4 cm^−1^, and the spectra of the samples were obtained. The FT‐IR spectra of the barley flour sample were recorded from 400 to 4000 cm^−1^ as shown in Figure 2. From the results, it was shown that the peak at 3244 cm^−1^ gives a medium and sharp appearance with O–H stretching vibration, which indicates the presence of an alcoholic group. There was a wide spectrum obtained from 2000 to 2400 cm,^−1^ while the strong absorption peak was obtained at 2381 cm^−1^. A shift in the band, however, indicates the presence of the carbon dioxide group and results in a change in the functional group at 2381 cm^−1^ with O=C=O stretching. The band from 1800 cm^−1^ fell down and formed narrow spectra.

The medium peak at 1375 cm^−1^ forms the CH_3_ bending, which indicates the presence of an alkane group. The strong peak obtained from 1000 to 1300 cm^−1^ indicates the presence of an esterified carboxylic group (‐COOR) and a glycosidic bond (C‐O). Moreover, the absorption peak at 1047 cm^−1^ represents the stretching vibration of CO˗O˗CO, which represents an anhydride group in the barley flour ( *H. vulgare* ). The outcomes of our investigation were consistent with those of Zhu et al. (2020), who investigated the functional groups found in barley flours from the highlands. Similarly, the findings of our investigation were very similar to those of Singh et al. (2024) study, which investigated the FTIR spectra of nonconventional pasta made from barley.

##### 3.1.4.2 FTIR of Pearl Millet Samples

**FIGURE 3:**
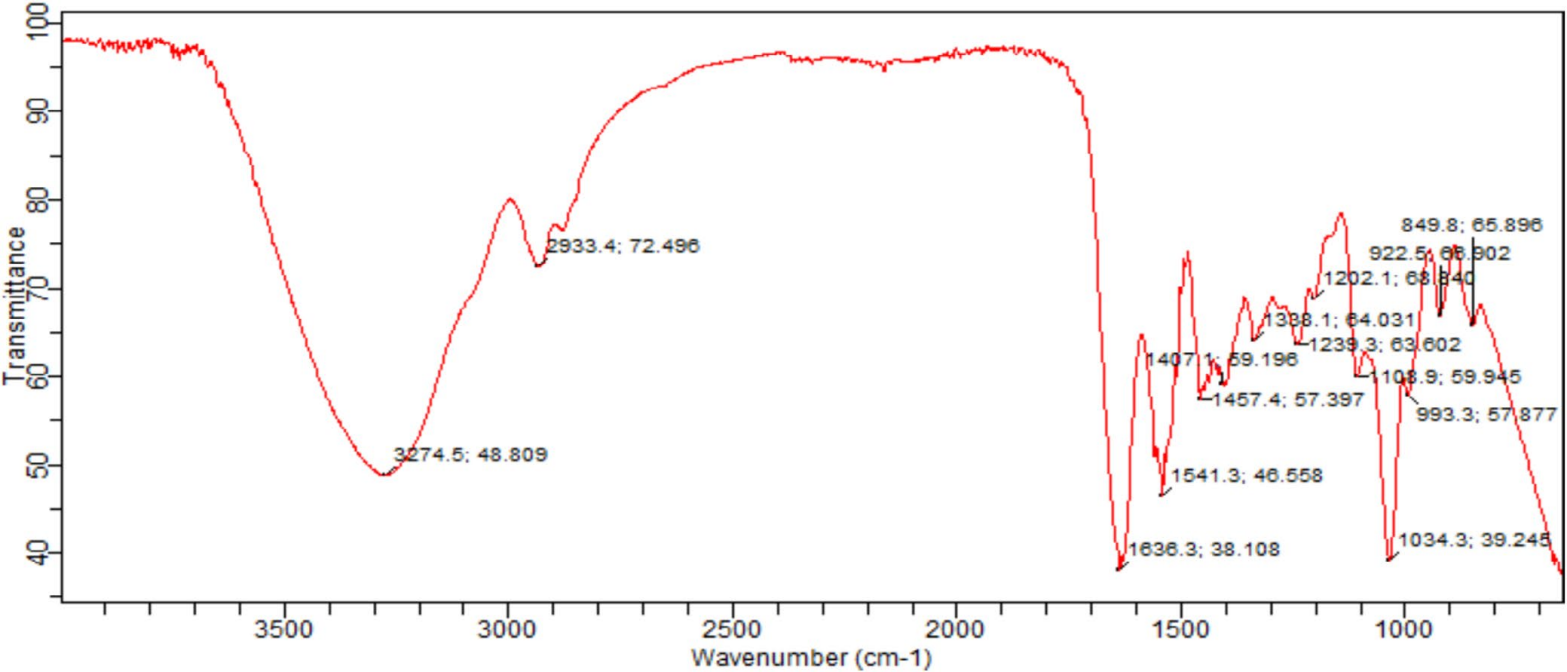
FTIR Spectra of Pearl millet ( *P. glaucum* ) flour.

The broad absorption band at 3500 cm^−1^, which is connected to O‐H stretching and indicates the existence of an alcohol group, was visible in the pearl millet flour FTIR spectra, as shown in Figure 3.

The absorption band at 2900 cm^−1^ is associated with the functional group alkane with C‐H stretch. Then, amplification of the intensities between 1500 cm^−1^ and 1200 cm^−1^ showed the existence of numerous absorption bands found, which were allocated to C‐H bending, asymmetric O‐H bending, and C‐O‐ asymmetric stretching in the pearl millet flour ( *P. glaucum* ) and indicates the presence of alcohol and carboxylic group. Table 3 shows the FTIR spectrum waves, vibration types, and functional groups. Prior research on the functional and microstructural characteristics of pearl millet was conducted by Gull et al. (2016), and their findings aligned with current results.

**TABLE 3:** FTIR spectrum waves, vibration types, and functional groups.

Sr. No	Wave No. cm^−1^	Vibration type	Functional group
1	3500	O˗H Stretching	Alcohol
2	2400	O=C=O Stretching	Carbon dioxide
3	2000	C˗H Bending	Aromatic compounds
4	2275	N=C=O Stretching	Nitrile
5	810	C˗H Bending	1,4‐disubstituted or tetra substituted
6	790	C=C Bending	Alkene
7	690	C˗Br Stretching	Halo compounds

##### 3.1.4.3 Scanning Electron Microscopy (SEM)

Scanning electron microscopy (SEM) has emerged as a crucial instrument. SEM is one of the most significant microscopy methods and is widely used because of its potential for high‐resolution topography of the surface imaging of bulk samples (Ali et al. 2023). Additionally, various studies have demonstrated that the SEM mode of transmission has possibilities for the study of nanomaterials (Xu et al. 2020). SEM, which provides images with a 5,00,000× magnification and resolutions better than 1 nm, is used to describe the appearance and crystallography of both organic and inorganic substances (Ganesh 2022). It uses an electron beam that contacts the substance in order to produce electrons called secondary electrons (SE) and backscattered electrons (BSE), which are then picked up by monitors (Datye and DeLaRiva 2023).

**FIGURE 4:**
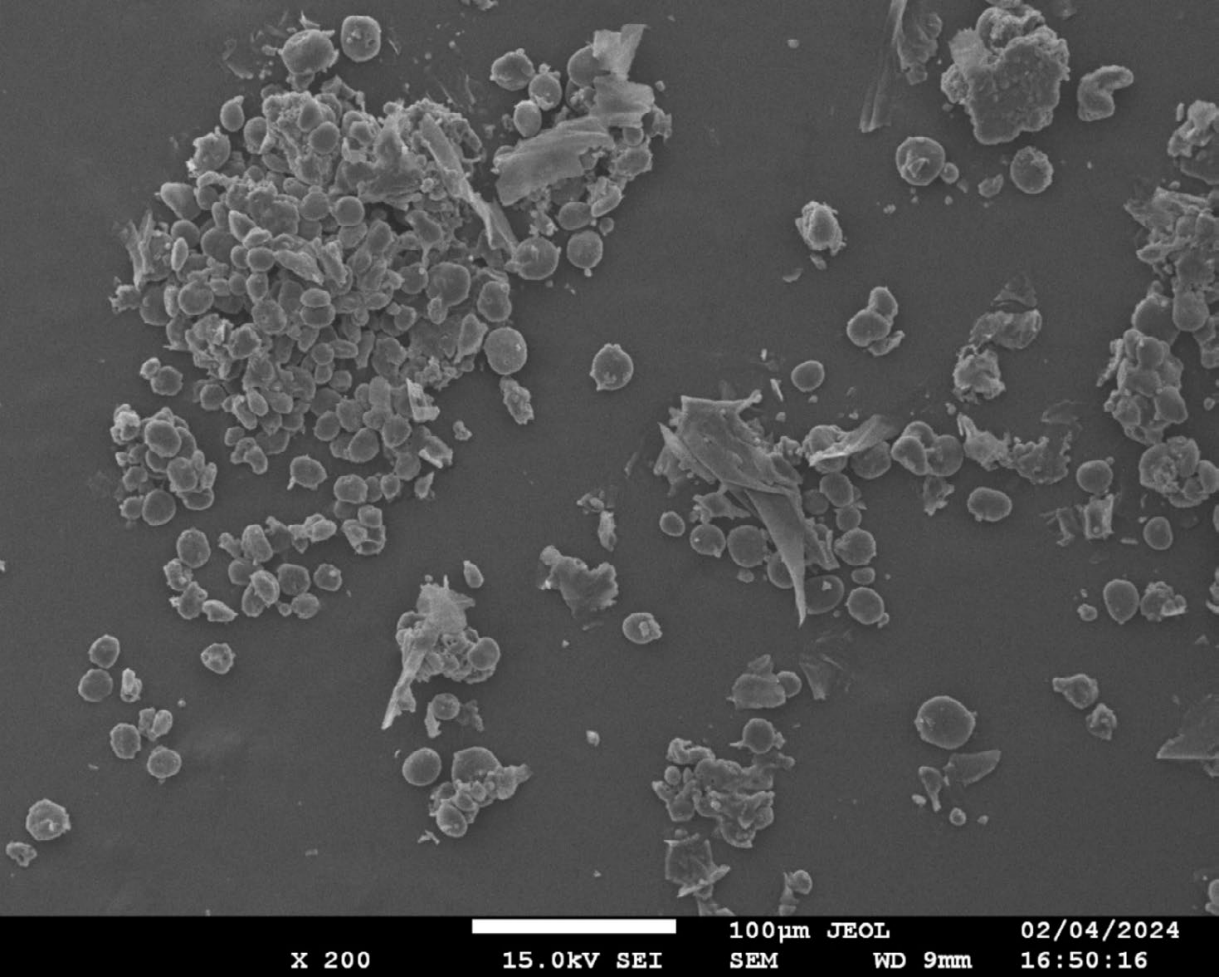
Morphological view of Barley ( *H. vulgare* ).

**FIGURE 5:**
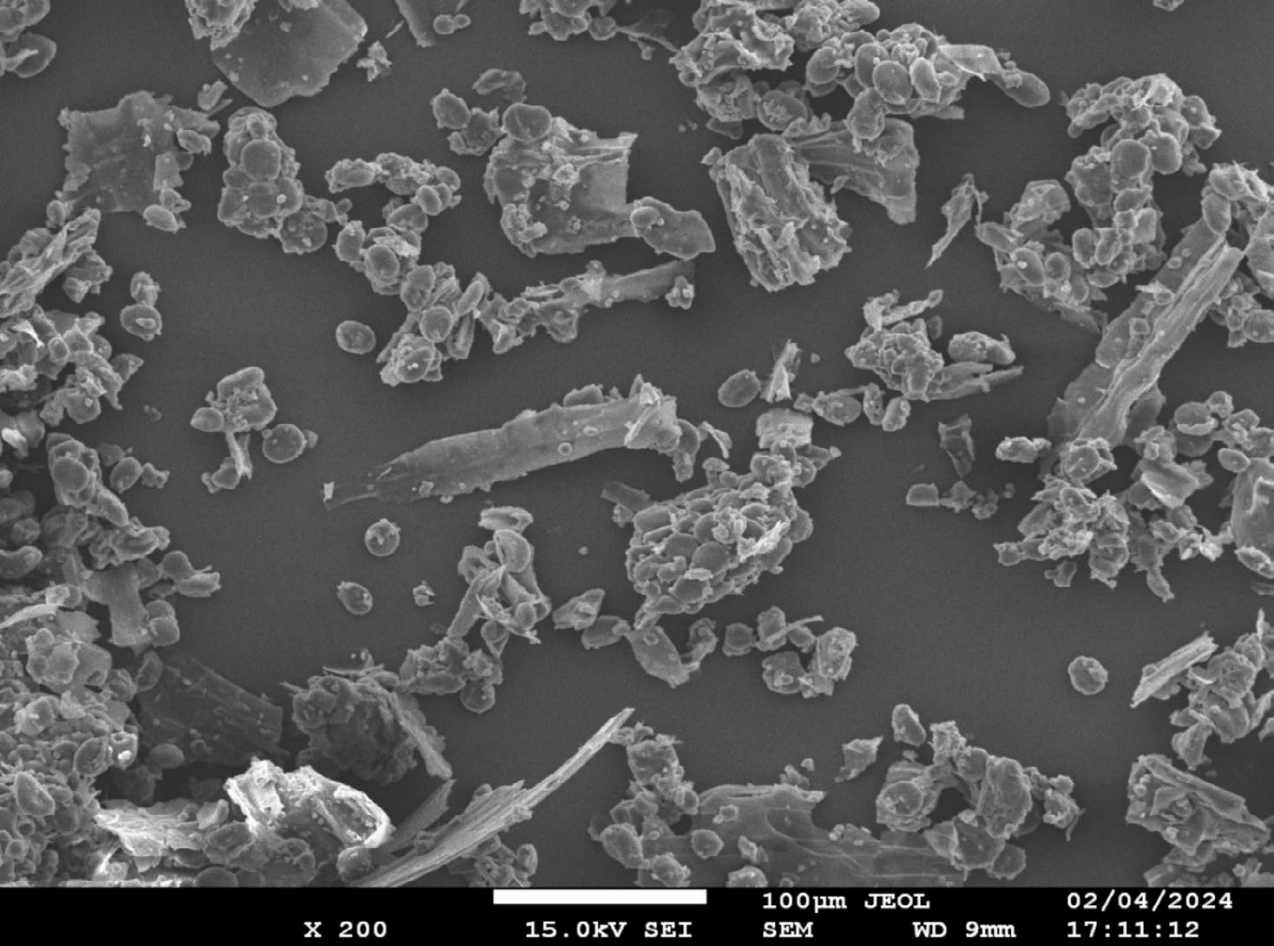
Morphological view of Pearl Millet ( *P. glaucum* ).

With this method, electrons with high energy are allowed to leave the specimen's surface and can be used to explore the nanomaterial's properties on a small scale. Barley displayed smaller, spherical, circular granules with smooth edges and irregular protein bodies connected to the surface, which represented a typical bimodal granule size distribution, as seen in Figure 4, based on the variations in the size and shape of non‐starch polysaccharides. On the other hand, a rough and wrinkled surface was seen in the instance of pearl millet flour. Additionally, as seen in Figure 5, the sample has a hollow and a cylindrical morphology with some voids. Moreover, SEM has become an increasingly useful tool for observing food microstructure, because it provides a detailed perspective on granule surface characteristics. The physicochemical characteristics and structural morphology of barley flour were evaluated by Zhao et al. (2022) and the outcomes of this investigation were consistent with our current findings.

##### 3.1.4.4 Extraction of Arabinoxylan and β‐Glucan

The effectiveness of extracting particular components, like fiber or bioactive chemicals, from the raw material is typically evaluated as extraction yield, which measures the quantity of the desired component attained from the raw material relative to the initial quantity. The extraction process, environmental parameters, and raw material characteristics are the variables that may affect the yield. Mean values of arabinoxylan and β‐glucan, starch, protein, and other carbohydrates (i.e., galactose, mannose, and fructose) composition in barley and pearl millet flour fractions and their water extracts are presented in Table 4. Mean values of extraction yield of AX in barley and pearl millet were (7.38% ± 0.06% and 3.51% ± 0.08%), respectively.

**TABLE 4:** Mean values for extraction yield.

Varieties	Yield AX%	Ara%	Xyl%	Man%	Gal%	Glc%	ARA/XYL%	Ferulic acid/Uronic acid%
Barley	7.38 ± 0.06^a^	45.99 ± 0.03^a^	47.78 ± 0.13^a^	0.73 ± 0.01^b^	3.91 ± 0.04^b^	1.42 ± 0.04^a^	0.67 ± 0.01^b^	0.93 ± 0.03^b^
Pearl millet	3.51 ± 0.08^b^	38.13 ± 0.24^b^	35.95 ± 0.19^b^	3.5 ± 0.14^a^	15.91 ± 0.15^a^	0.52 ± 0.07^b^	1.03 ± 0.02^a^	2.35 ± 0.04^a^

*Note:* The values are expressed as mean ± standard deviation of triplicate values.

However, mean values of other compositional fractions of barley flour ( *H. vulgare* ) Arabinose%, Xylose%, Mannose%, Galactose%, Glucose%, Arabinose/Xylose ratio%, Proteins%, and Ferulic acid% were (45.99 ± 0.03), (47.78 ± 0.13), (0.73 ± 0.01), (3.91 ± 0.04), (1.42 ± 0.04), (0.67 ± 0.01), (12.76 ± 0.17), and (0.93 ± 0.03), respectively. Mean values for extraction yield of pearl millet ( *P. glaucum* ) showed the presence of Arabinose (38.13% ± 0.24%), Xylose (35.95% ± 0.19%), Mannose (3.5% ± 0.14%), Galactose (15.91% ± 0.15%), Glucose (0.52% ± 0.07%), Arabinose/Xylose ratio (1.03% ± 0.02%), Proteins (14.86% ± 0.66%) and Uronic acid (2.35% ± 0.04%), respectively. Barley (Talbina‐21) has significantly higher arabinoxylan yield (AX%), arabinose (Ara%), xylose (Xyl%), and glucose (Glc%), making it richer in dietary fiber and structural carbohydrates. Pearl Millet (Shahansha) dominates in mannose (Man%), galactose (Gal%), ARA/XYL ratio, and ferulic/uronic acid, suggesting stronger antioxidant properties and more branched hemicellulose. The differences highlight barley's superior fiber content for gut health, while pearl millet offers better antioxidant activity and unique polysaccharide structures.

Ferulic acid and uronic acid present in plant‐derived cereals barley flour ( *H. vulgare* ) and pearl millet ( *P. glaucum* ) offer antioxidant benefits, protecting cells from oxidative stress and potentially reducing the risk of cardiovascular diseases. Additionally, they also exhibit anti‐inflammatory effects, support gut health, and possess anticancer properties (Chaudhary et al. 2019; Alam 2019; Erdmann et al. 2021). Thus, the results of our current research are in line with the previous findings of Kaur et al. (2023) who worked on the green extraction of dietary fibers from millet bran, and results are comparable for the extraction yield of raw material.

## 4 Conclusion

Results obtained revealed significant differences between the chemical composition of dried barley and pearl millet. Pearl millet had higher moisture (11.20% ± 0.50%) and ash (2.68% ± 0.02%) content, whereas barley had higher protein (13.60% ± 0.02%) and fiber (4.77% ± 0.01%) content. Pearl millet also had higher fat (4.87% ± 0.21%) and nitrogen‐free extract (NFE) (67.20% ± 0.14%). Mineral composition analysis showed barley had higher potassium (142.53 ± 3.18 mg/100 g) and phosphorus (350.58 ± 1.39 mg/100 g), whereas pearl millet had higher potassium (306.33 ± 3.2 mg/100 g) and iron (7.81 ± 0.05 mg/100 g). Both cereals contained essential minerals like calcium, magnesium, and sodium. Antioxidant activity measured through FRAP and DPPH tests indicated higher antioxidant activity in barley for DPPH (105.72 ± 0.02 mg GAE/g), while pearl millet showed higher activity for FRAP (5.22 ± 0.04 TE/g). Barley also had higher total phenolic and flavonoid contents. The compositional analysis of the flours revealed the presence of various sugars, proteins, ferulic acid, uronic acid, and dietary fiber components (arabinoxylan & β‐glucan). β‐glucan and arabinoxylan find numerous uses in food formulations, where they improve sensory and physical qualities in addition to nutritional value. This leads to better food options and meets the growing need for functional foods. However, to prove the biological usefulness of dietary arabinoxylan & β‐glucan in human health intervention, a variety of bioassays and studies, including in vitro assays, epidemiological (population surveys), in vivo (animal trials) and therapeutic (human intervention) experiments are required. This study provides insight and opens up new avenues of future research focusing on the development of nutrient‐rich functional foods prepared using genetically improved and functionally diverse grains, with significant benefits for human health and to attain food security and sustainable agriculture.

## Author Contributions

**Iqra Khalil:** methodology (equal), writing – original draft (equal), writing – review and editing (equal). **Shahid Bashir:** project administration (equal), supervision (equal), writing – original draft (equal). **Kanza Saeed:** methodology (equal), validation (equal), writing – review and editing (equal). **Tawfiq Alsulami:** formal analysis (equal), validation (equal), writing – review and editing (equal). **Hamad Rafique:** formal analysis (equal), writing – review and editing (equal). **Emery Kasongo Lenge Mukonzo :** methodology (equal), writing – review and editing (equal).

## Disclosure

The authors have nothing to report.

## Consent

All authors agree to publish.

## Data Availability

The authors confirm that the data supporting the findings of this study are available within the article.
